# Supplementary material for: Single-cell chromatin accessibility and transcriptomic characterization of Behcet’s disease
Source: Commun Biol. 2023 Oct 17;6:1048. doi: 10.1038/s42003-023-05420-x (PMC10582193; doi:10.1038/s42003-023-05420-x)
Supplement: Supplementary file 2 — Supplementary Information [file 42003_2023_5420_MOESM2_ESM.pdf]

Supplementary Figures

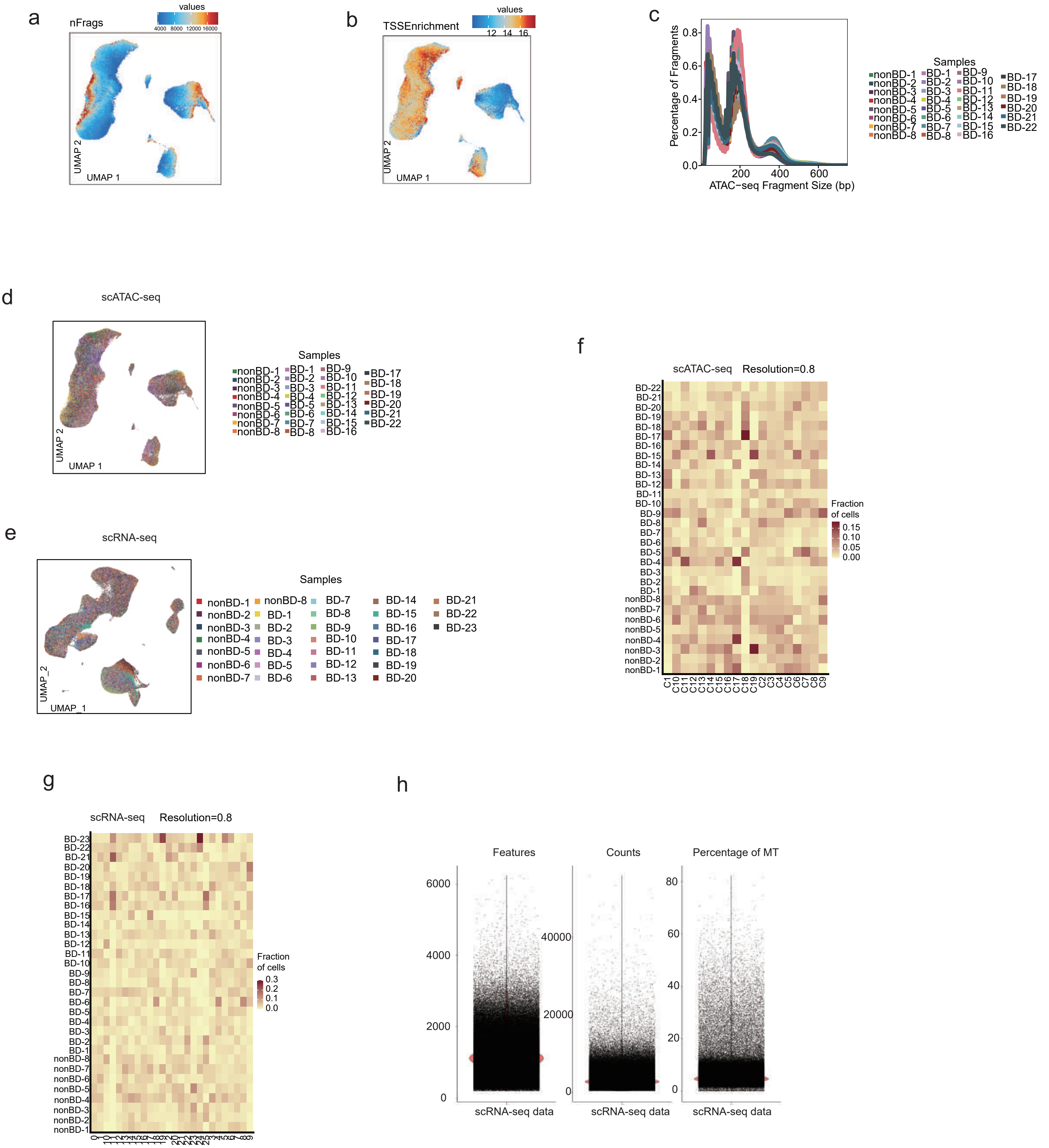

Supplementary Figure 1

Supplementary Figure 1. Quality control of scATAC-seq and scRNA-seq data.

a. UMAP projection of scATAC-seq cells colored by fraction of reads in peaks. **b.** UMAP projection of scATAC-seq cells colored by total number of unique nuclear Tn5 insertion fragments. **c.** Aggregated scATAC-seq fragment size distributions across individual experiments demonstrating sub-, mono- and multi nucleosome spanning ATAC-seq fragments. **d.** UAMP projection of scATAC-seq cells colored by donors. **e.** UAMP projection of scRNA-seq cells colored by donors. **f.** Heatmap of confusion matrix showing the percent of cells from each sample and each cluster from scATAC-seq dataset. **g.** Heatmap of confusion matrix showing the percent of cells from each sample and each cluster from scATAC-seq dataset. **h.** Violin plot of the features, counts, and percentage of mitochondrial genes in scRNA-seq dataset. All data are aligned and annotated to hg38 reference genome.

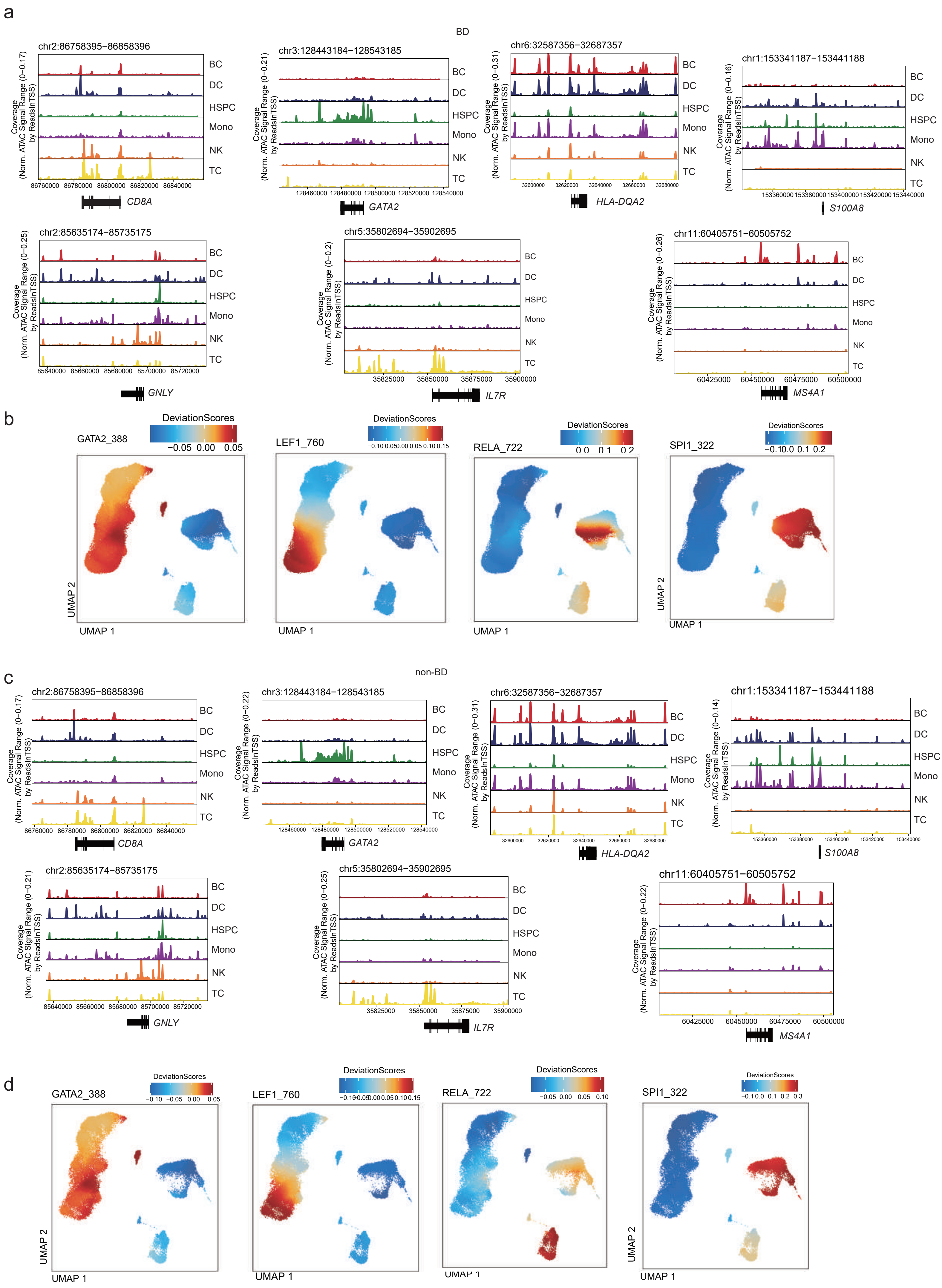

Supplementary Figure 2

Supplementary Figure 2. Separate chromatin analysis of BD and nonBD groups.

a. Accessibility profiles in the BD group for scATAC-seq six main cell type at canonical cell-type marker genes. **b.** UMAP projection of scATAC-seq peripheral blood profiles in the BD group colored by chromVAR TF motif bias-corrected deviations for the indicated factors. **c.** Accessibility profiles in the non-BD group for scATAC-seq six main cell type at canonical cell-type marker genes. **d.** UMAP projection of scATAC-seq peripheral blood profiles in the non-BD group colored by chromVAR TF motif bias-corrected deviations for the indicated factors. All data are aligned and annotated to hg38 reference genome.

a

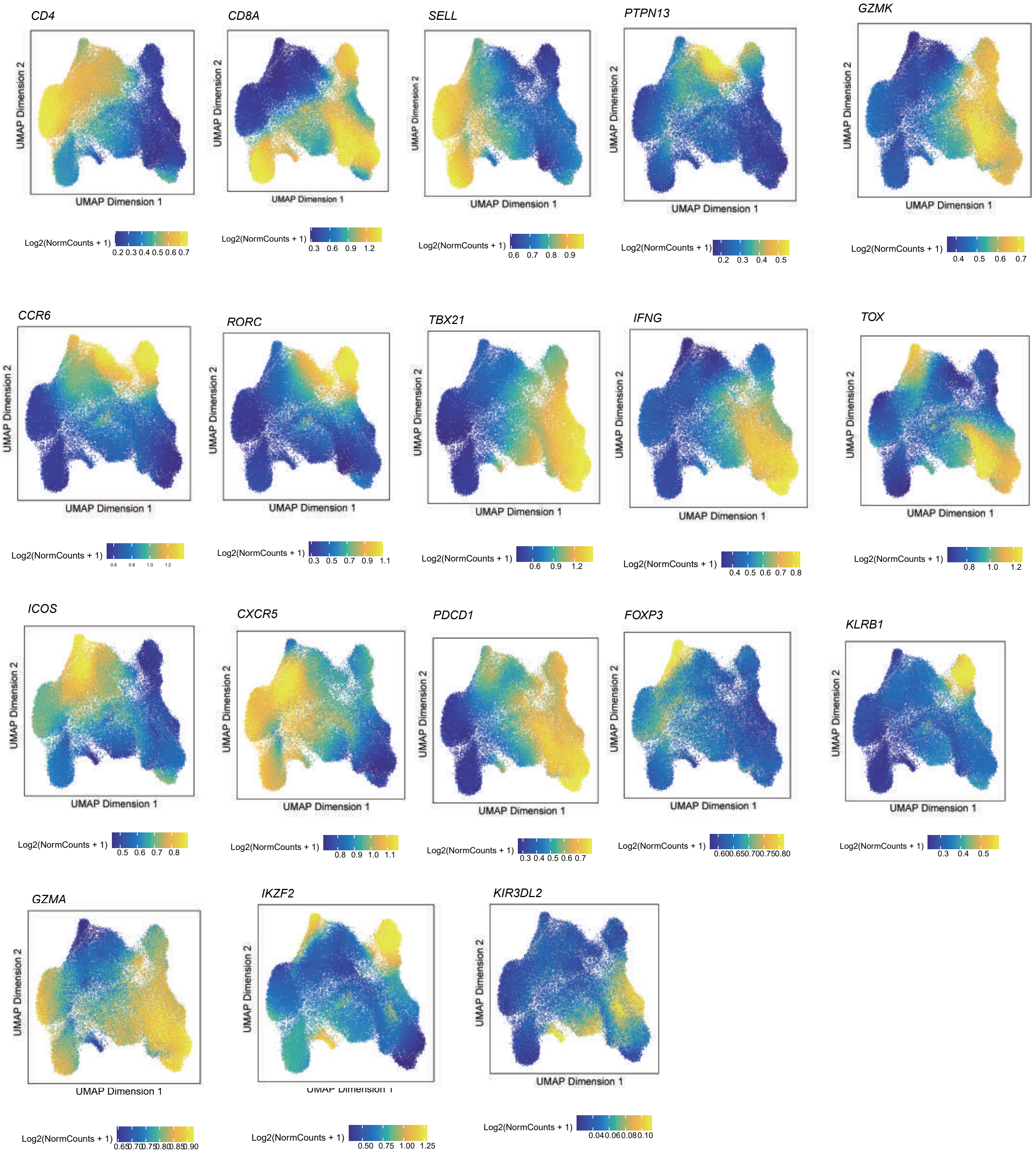

b

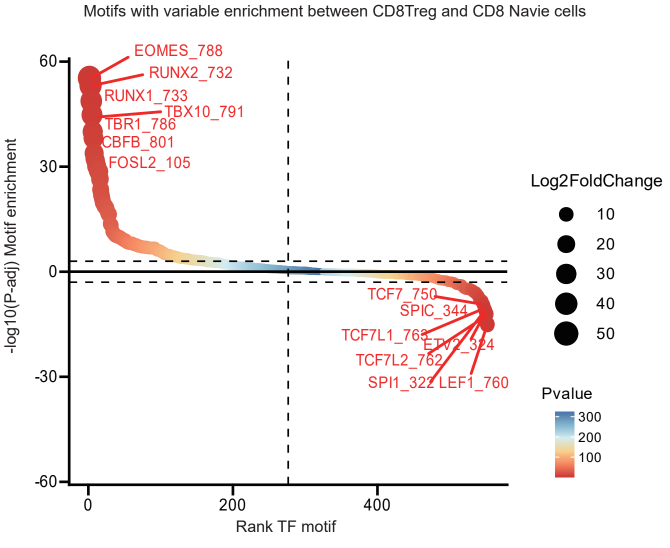

Supplementary Figure 3

Supplementary Figure 3. Validation of T cell marker genes for scATAC-seq dataset.
a. UMAP projection colored by gene activity scores for the annotated lineage-defining genes in non-BD group of scATAC-seq dataset. The minimum and maximum gene activity scores are shown in each panel. **b.** FDR-corrected P values from two-sided, two-sample t-tests of differential ArchR motif enrichment comparison between CD8 naive and CD8 Treg cells for transcription factor motifs. All data are aligned and annotated to hg38 reference genome.

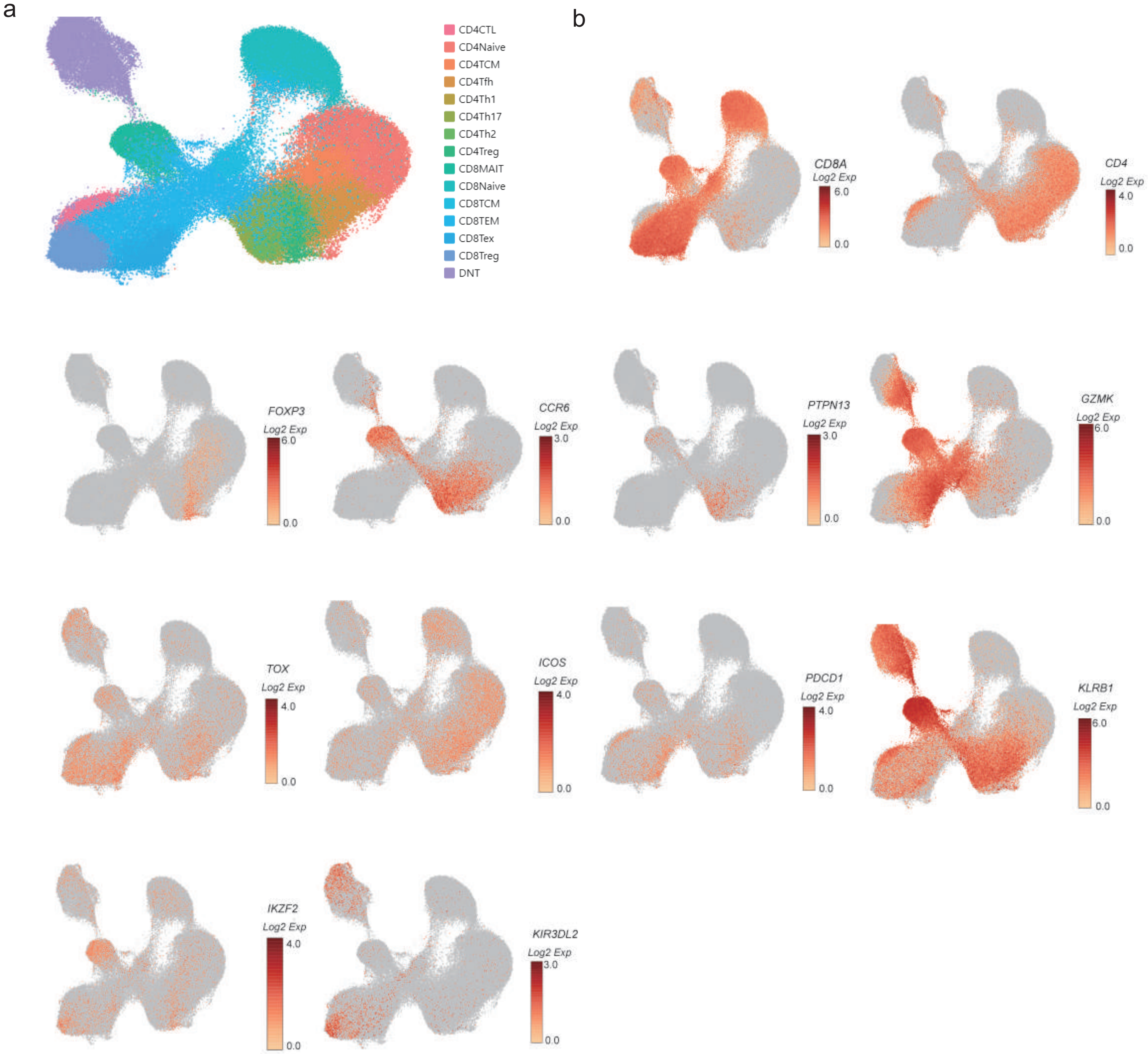

Supplementary Figure 4

Supplementary Figure 4. Validation of T cell marker genes for scRNA-seq dataset.

a. UMAP projection of T cell profiles of peripheral blood immune cell types in scRNA-seq dataset. Dots represent individual cells, and colors indicate T cell subpopulation cell types. **b.** UMAP projection colored by log-normalized gene expression to the indicated gene. All data are aligned and annotated to hg38 reference genome.

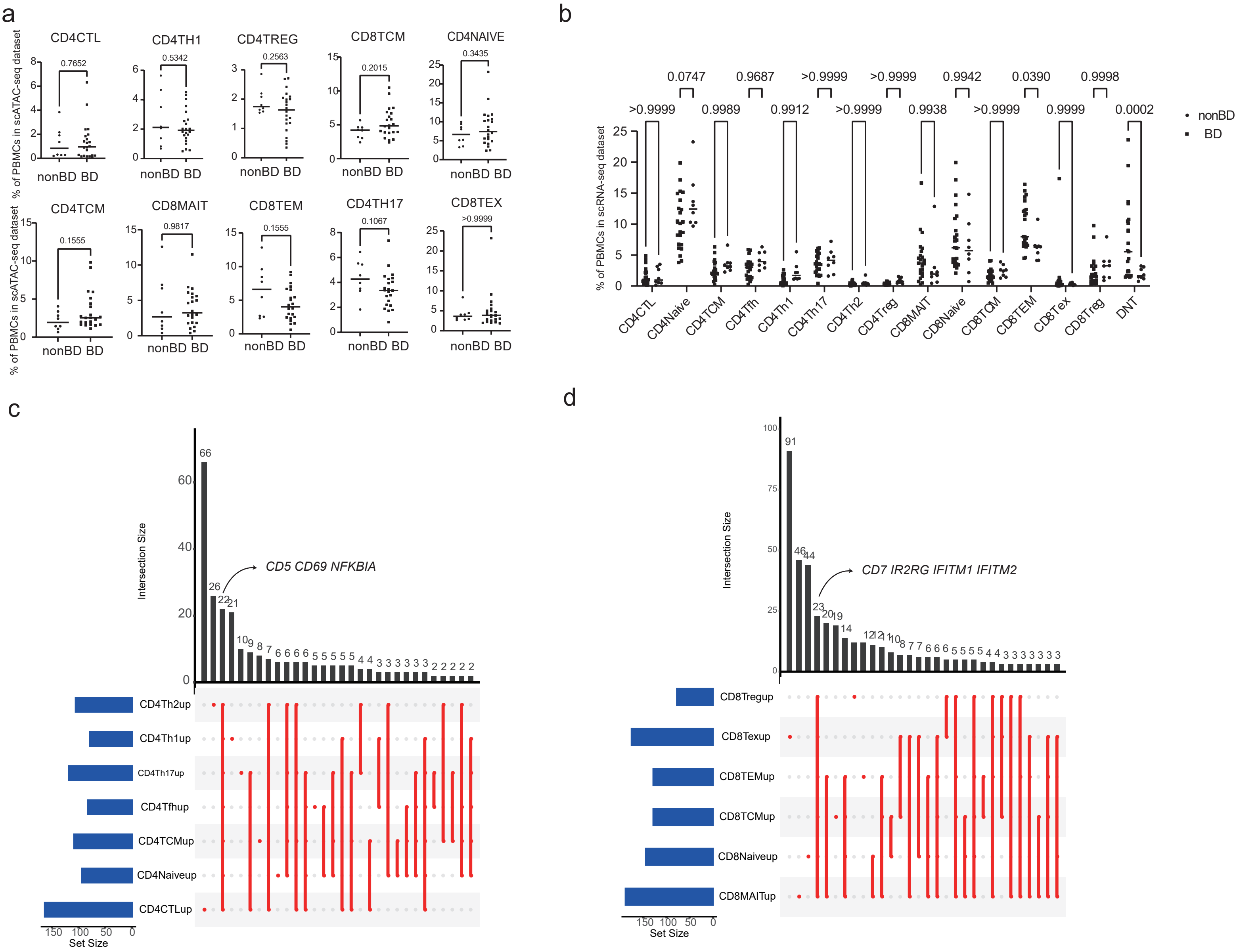

Supplementary Figure 5

Supplementary Figure 5. Changes in T cell subsets of scATAC-seq and scRNA-seq dataset among non-BD and BD.

a. Differences in the proportions of indicated T cell subsets in scATAC-seq dataset among non-BD (n = 8) and BD groups (n = 22). The p values were calculated using two-sided Wilcoxon rank-sum test. The horizontal lines denote median. **b.** Differences in the proportions of indicated T cell subsets in scRNA-seq dataset among non-BD (n = 8) and BD groups (n = 23). The p values were calculated using two-sided Wilcoxon rank-sum test. The horizontal lines denote median. **c.** UpSet plot showing the integrated comparative analysis of upregulated differentially expressed genes (DEGs) in CD4 T cells between non-BD and BD groups. Upregulated DEGs: upregulated in BD, downregulated in non-BD. The count showing the number of DEGs. **d.** UpSet plot showing the integrated comparative analysis of upregulated DEGs in CD8 T cells between non-BD and BD groups. Upregulated DEGs: upregulated in BD, downregulated in non-BD. The count showing the number of DEGs. All data are aligned and annotated to hg38 reference genome.

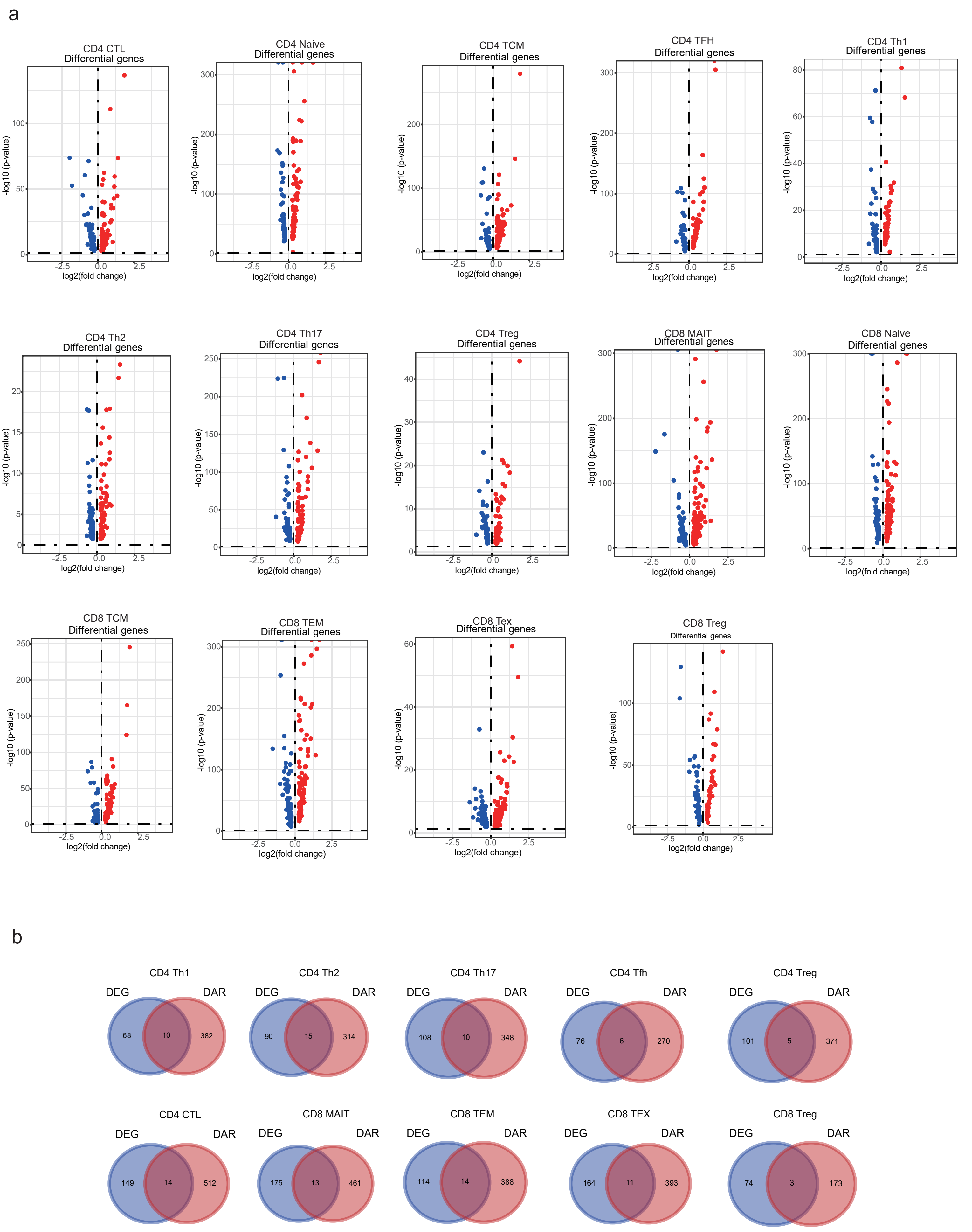

Supplementary Figure 6

Supplementary Figure 6. Chromatin and genetic changes in T cell subsets of scATAC-seq and scRNA-seq dataset among non-BD and BD.

a. Volcano plots of DEGs distribution between nonBD and BD groups in T cell subsets. **b.** Venn diagrams for each T cell subset showing the overlaps between the nearest genes of differential accessible chromatin regions in the BD group (DARs) and DEGs in the BD group. All data are aligned and annotated to hg38 reference genome.

a

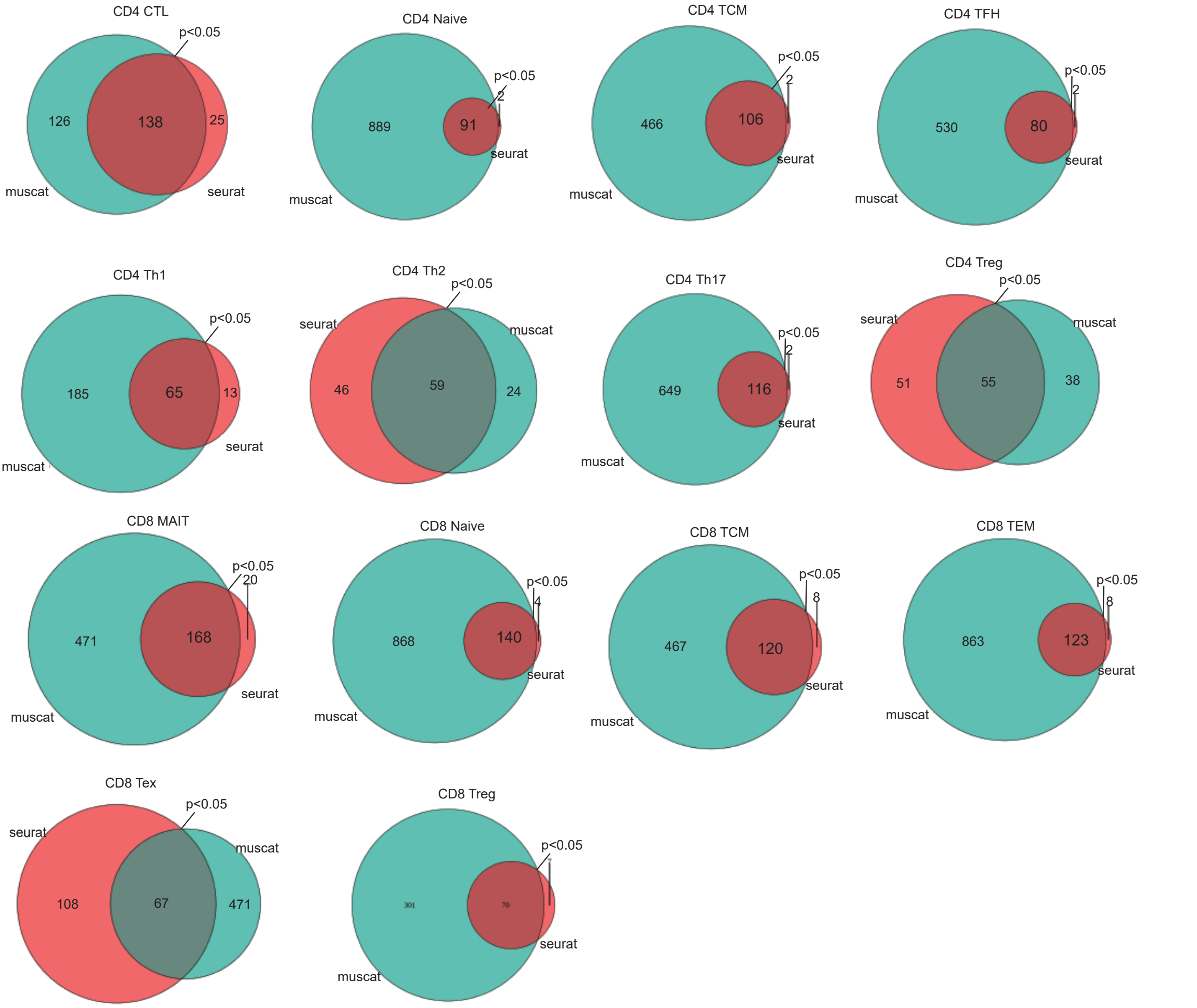

b

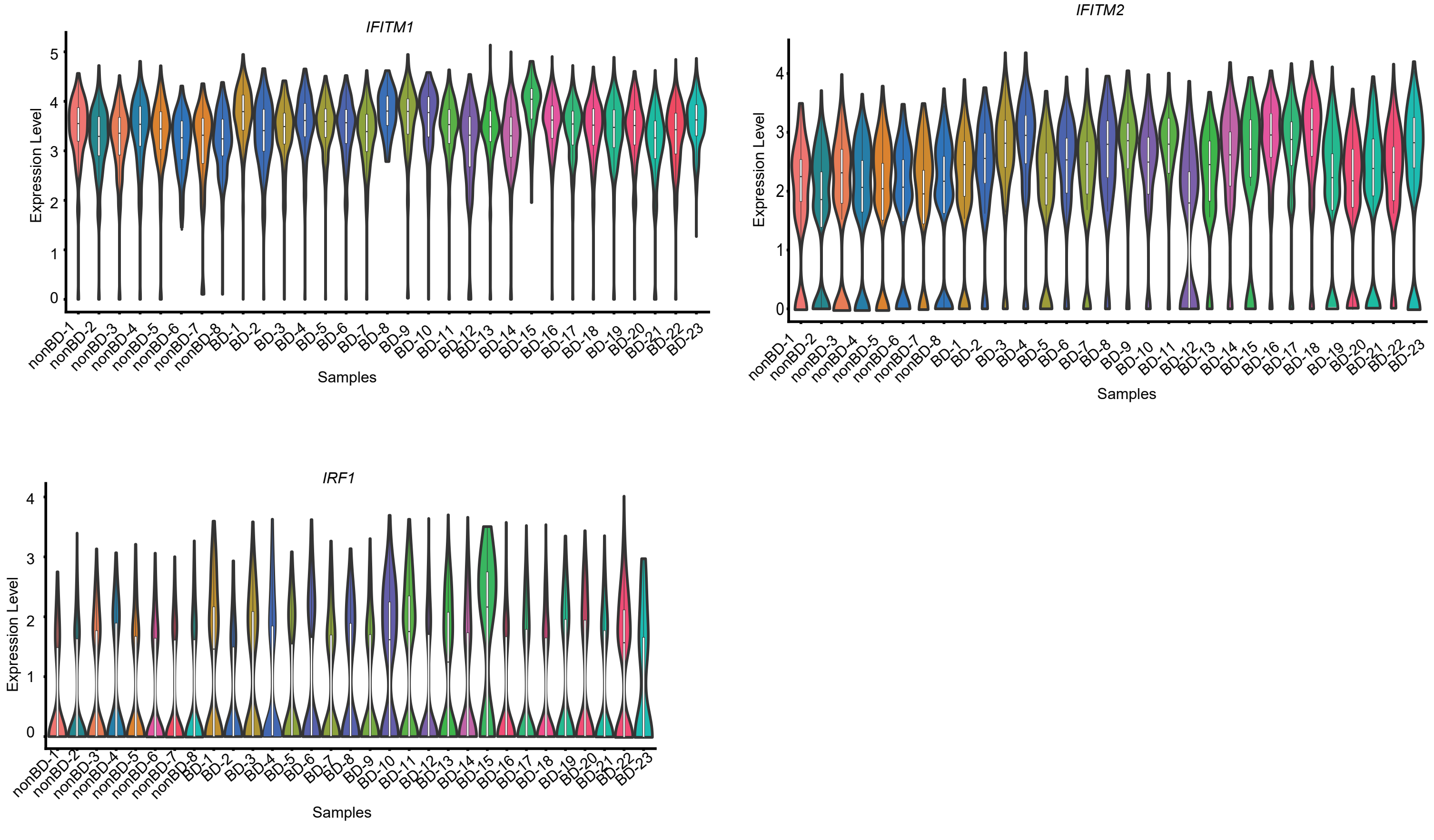

Supplementary Figure 7

Supplementary Figure 7. Validation of genetic changes in T cell subsets of the scRNA-seq dataset among non-BD and BD.

a. Venn diagrams for each T cell subset showing the overlaps between the DEGs calculated by *muscat* and the DEGs calculated by *Seurat*. A one-sided Fisher's exact test was used for gene-set overlap significance. **b.** Violin boxplots of upregulated genes (interferon-induced genes) that enriched in interferon pathway in Th17 cells separated by each donor. The top lines of the boxes denote the third quartile. The bottom lines of the boxes denote the first quartile. The horizontal lines of the boxes denote the median. All data are aligned and annotated to hg38 reference genome.

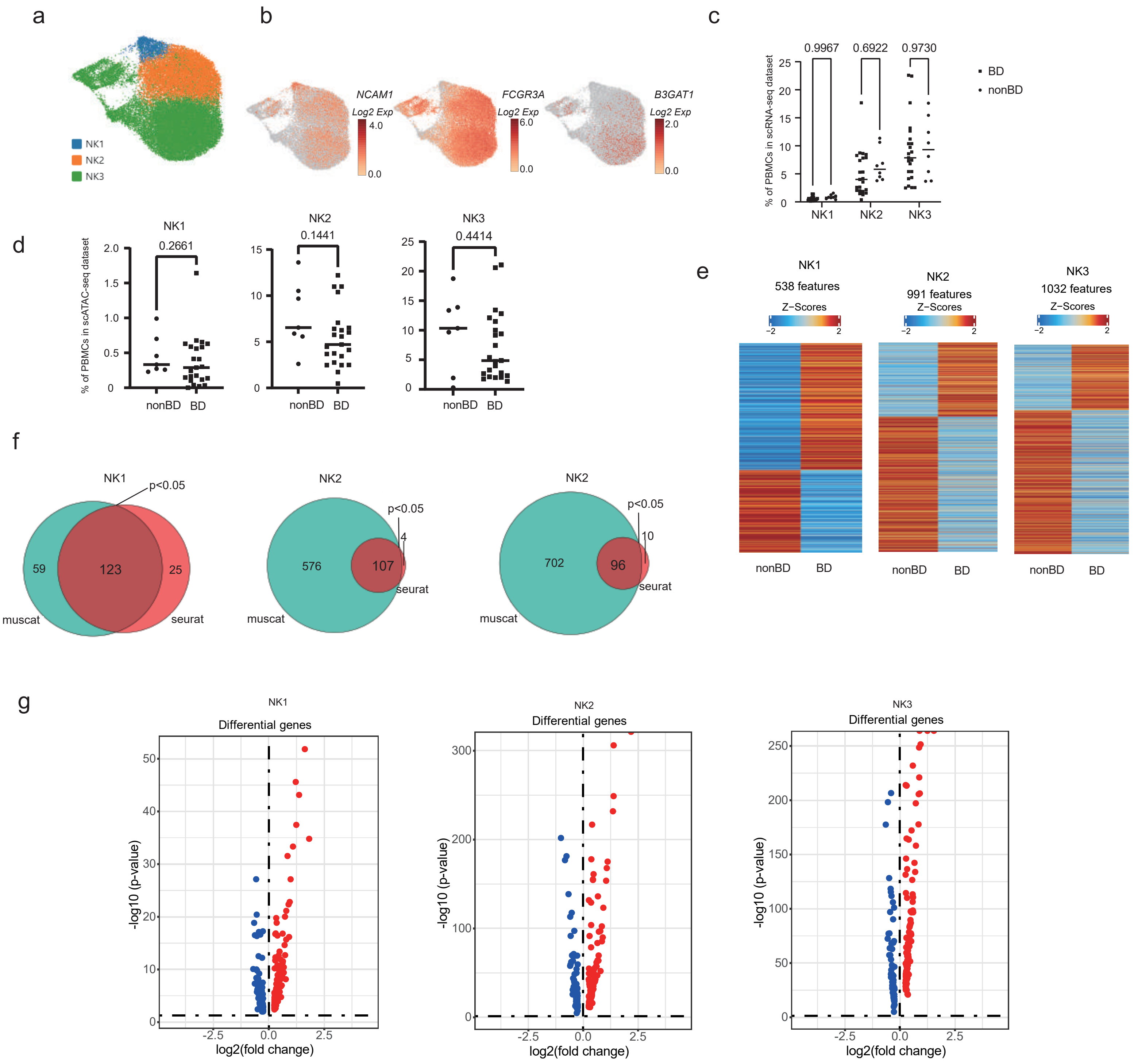

Supplementary Figure 8

Supplementary Figure 8. Changes in NK cell subsets of scATAC-seq and scRNA-seq dataset among non-BD and BD.

a. UMAP projection of NK cell profiles of peripheral blood immune cell types in scRNA-seq dataset. **b.** UMAP projection of NK cells colored by log-normalized gene expression to the indicated gene. **c.** Differences in the proportions of NK cell subsets in scRNA-seq dataset among non-BD (n = 8) and BD groups (n = 23). The p values were calculated using two-sided Wilcoxon rank-sum test. The horizontal lines denote median. d. Differences in the proportions of NK cell subsets in scATAC-seq dataset among non-BD (n = 8) and BD groups (n = 22). The p values were calculated using two-sided Wilcoxon rank-sum test. The horizontal lines denote median. **e.** Heatmap of Z-scores of DARs in NK1, NK2 and NK3 from non-BD and BD. **f.** Venn diagrams for each NK cell subset showing the overlaps between the DEGs calculated by *muscat* and the DEGs calculated by *Seurat*. A one-sided Fisher's exact test was used for gene-set overlap significance. **g.** Volcano plots of DEGs distribution between non-BD and BD groups in NK cell subsets. All data are aligned and annotated to hg38 reference genome.

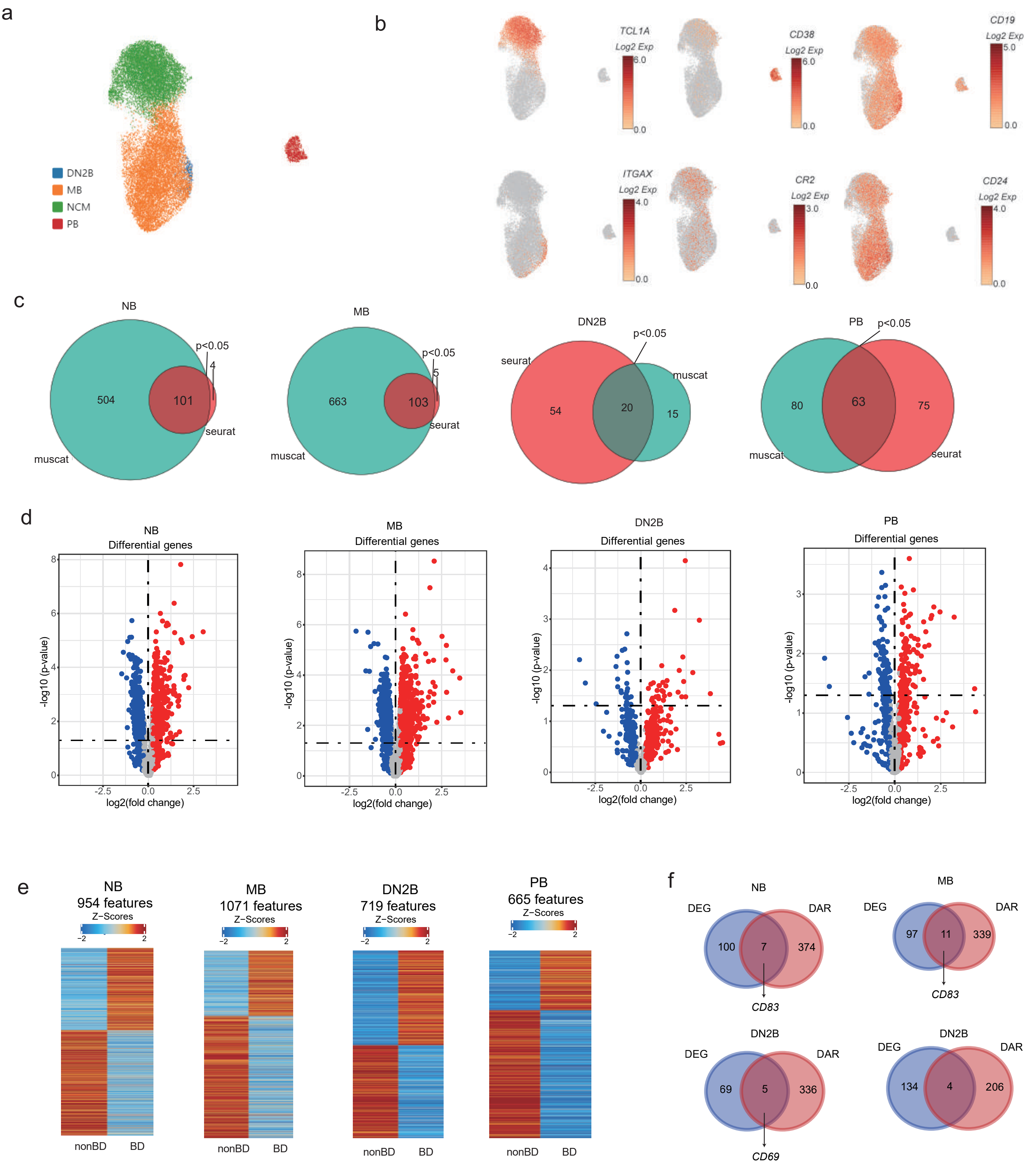

Supplementary Figure 9

Supplementary Figure 9. Changes in B cell subsets of scATAC-seq and scRNA-seq dataset among non-BD and BD.

a. UMAP projection of B cell profiles of peripheral blood immune cell types in scRNA-seq dataset. **b.** UMAP projection of B cells colored by log-normalized gene expression to the indicated gene. **c.** Venn diagrams for each B cell subset showing the overlaps between the DEGs calculated by *muscat* and the DEGs calculated by *Seurat*. A one-sided Fisher's exact test was used for gene-set overlap significance. **d.** Volcano plots of DEGs distribution between non-BD and BD groups in T cell subsets. **e.** Heatmap of Z-scores of DARs in B cell subsets from BD and non-BD groups. **f.** Venn diagrams for each major cell type showing the overlaps between the set of DARs and DEGs upregulated in that cell type. All data are aligned and annotated to hg38 reference genome.

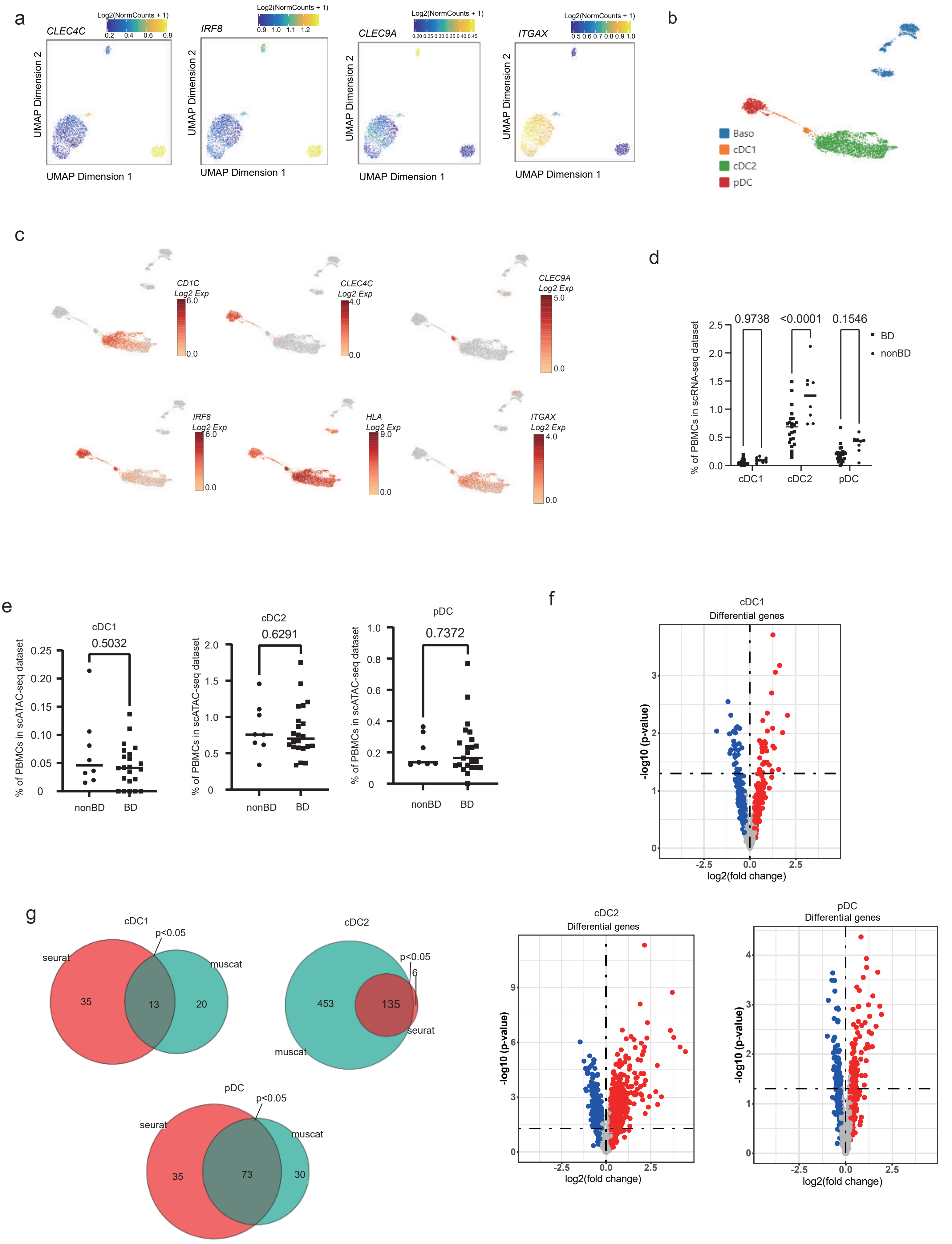

Supplementary Figure 10

Supplementary Figure 10. Validation DC marker genes and changes of DC for scRNA-seq and scATAC-seq dataset.

a. UMAP projection of DC colored by gene activity scores for the annotated lineage-defining genes of scATAC-seq dataset. The minimum and maximum gene activity scores are shown in each panel. **b.** UMAP projection of DC profiles of peripheral blood immune cell types in scRNA-seq dataset. **c.** UMAP projection of DC colored by log-normalized gene expression to the indicated gene. **d.** Differences in the proportions of indicated DC subsets in scRNA-seq dataset among non-BD (n = 8) and BD groups (n = 23). The p values were calculated using two-sided Wilcoxon rank-sum test. The horizontal lines denote median. e. Differences in the proportions of indicated DC subsets in scATAC-seq dataset among non-BD (n = 8) and BD groups (n = 22). The p values were calculated using two-sided Wilcoxon rank-sum test. The horizontal lines denote median. **f.** Volcano plots of DEGs distribution between non-BD and BD groups in DC subsets. g. Venn diagrams for each DC subset showing the overlaps between the DEGs calculated by *muscat* and the DEGs calculated by *Seurat*. A one-sided Fisher's exact test was used for gene-set overlap significance. All data are aligned and annotated to hg38 reference genome.

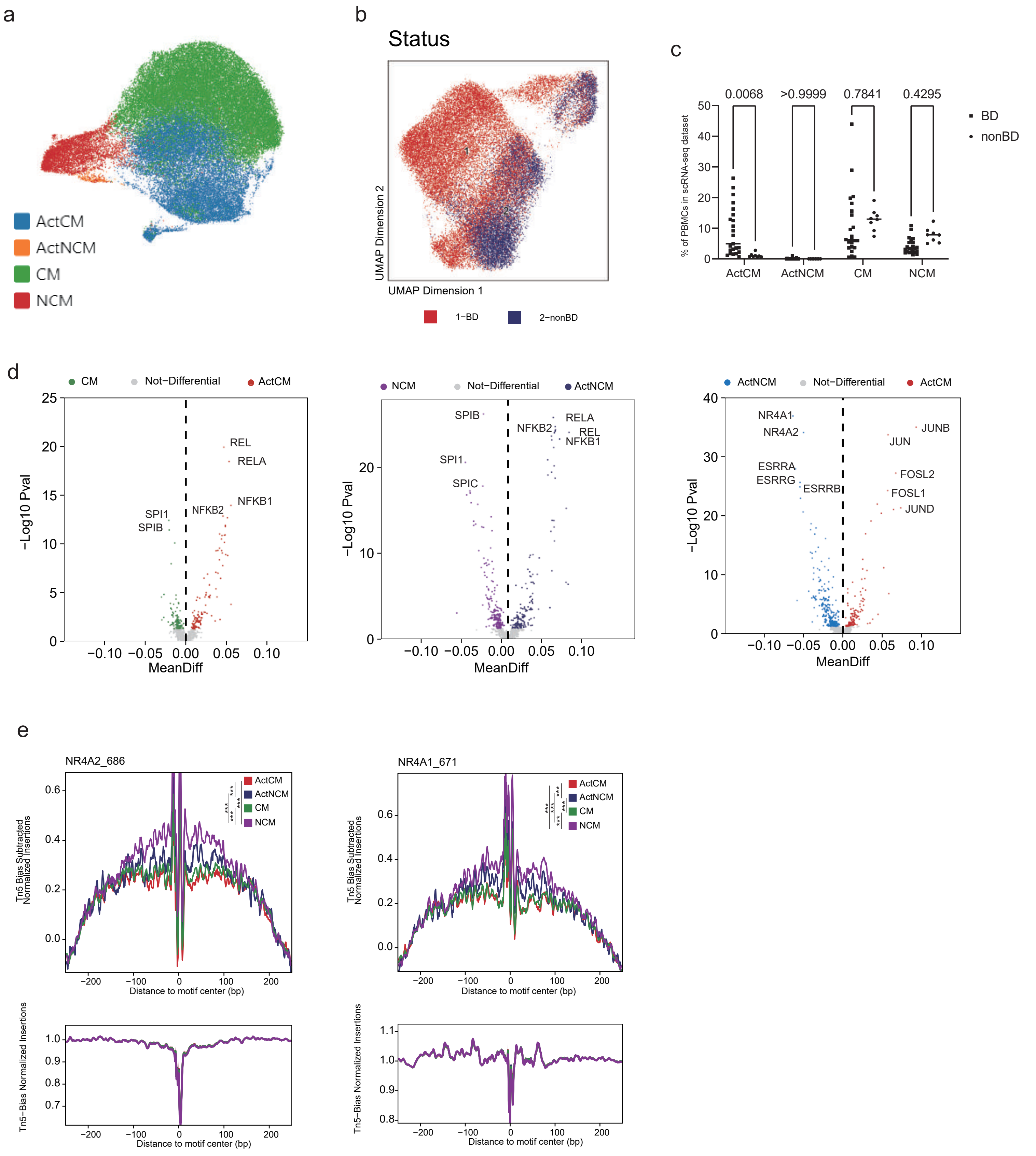

Supplementary Figure 11

Supplementary Figure 11. Changes of monocytes for scRNA-seq and scATAC-seq dataset.

a. UMAP projection of monocyte profiles of peripheral blood immune cell types in scRNA-seq dataset. **b.** Differences in the proportions of indicated monocytes subsets in scRNA-seq dataset among non-BD (n = 8) and BD groups (n = 23). The adjusted p values were calculated using two-sided pairwise Wilcoxon test. **c.** Differences in the proportions of indicated monocyte subsets in scRNA-seq dataset among non-BD (n = 8) and BD groups (n = 23). The p values were calculated using two-sided Wilcoxon rank-sum test. The horizontal lines denote median. **d.** Volcano plots showing the differential TF motif accessibility using the mean TF motif accessibility in the chromVAR TF bias-corrected deviation in ActCM vs CM, ActNCM vs NCM, and ActCM vs ActNCM. The p values were calculated using two-sided Wilcoxon rank-sum test. **e.** Comparison of aggregate TF footprints for NR4A1 and NR4A1 in monocyte subsets. TF footprint was compared by one-way ANOVA. All data are aligned and annotated to hg38 reference genome.

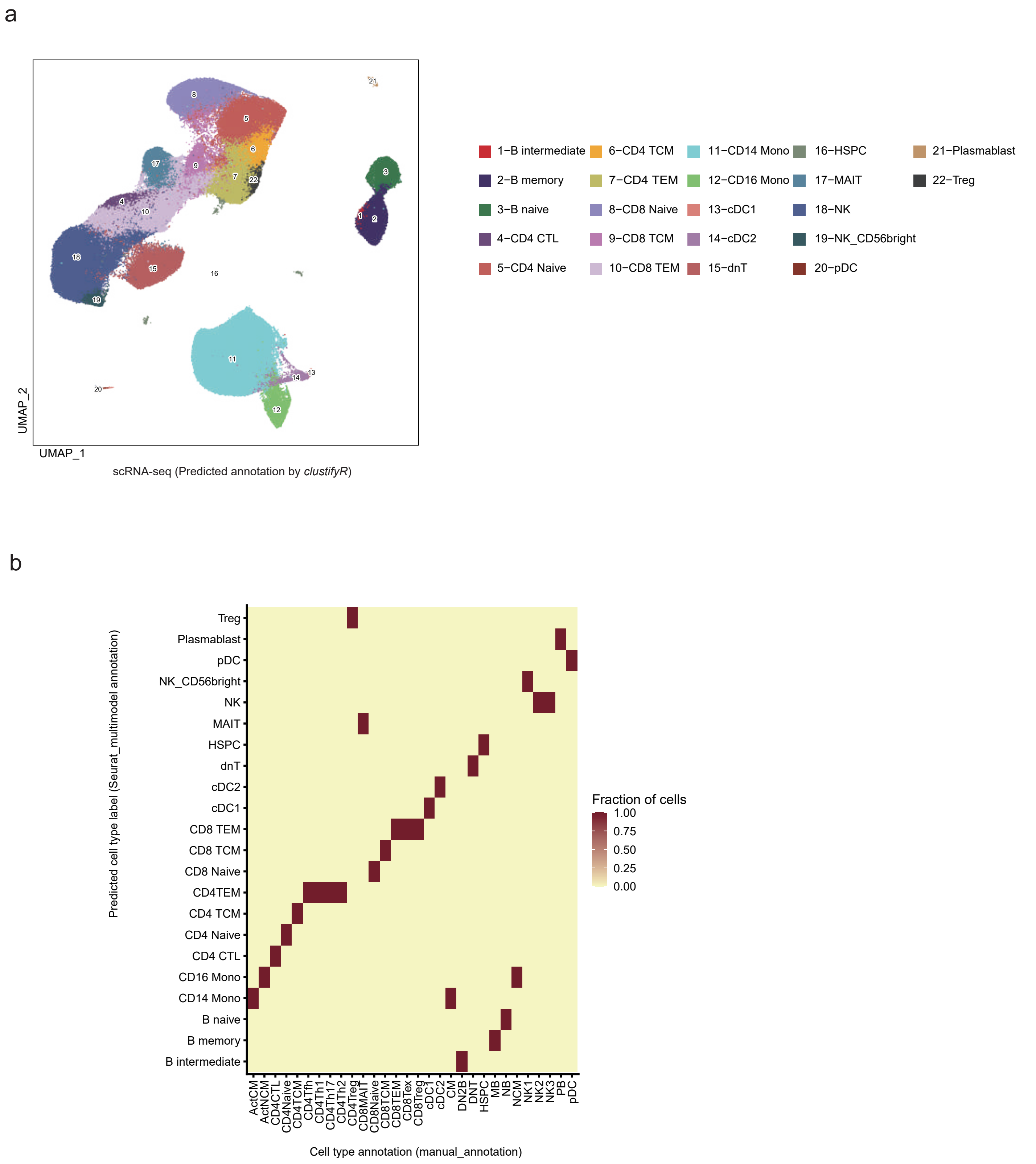

Supplementary Figure 12

Supplementary Figure 12. Validation of scRNA-seq cell type annotation using *clustifyR*.

a. UMAP projection of 22 cell clusters annotated by *clustifyR* using Seurat's multimodel annotation reference (colors) in PBMCs from our BD and non-BD scRNA-seq dataset. **b.** Heatmap showing the cell type alignment of Seurat's multi-model annotation reference and our manual cell type annotation. The color intensity represents the fraction of the assigned cells per cell type. All data are aligned and annotated to hg38 reference genome.

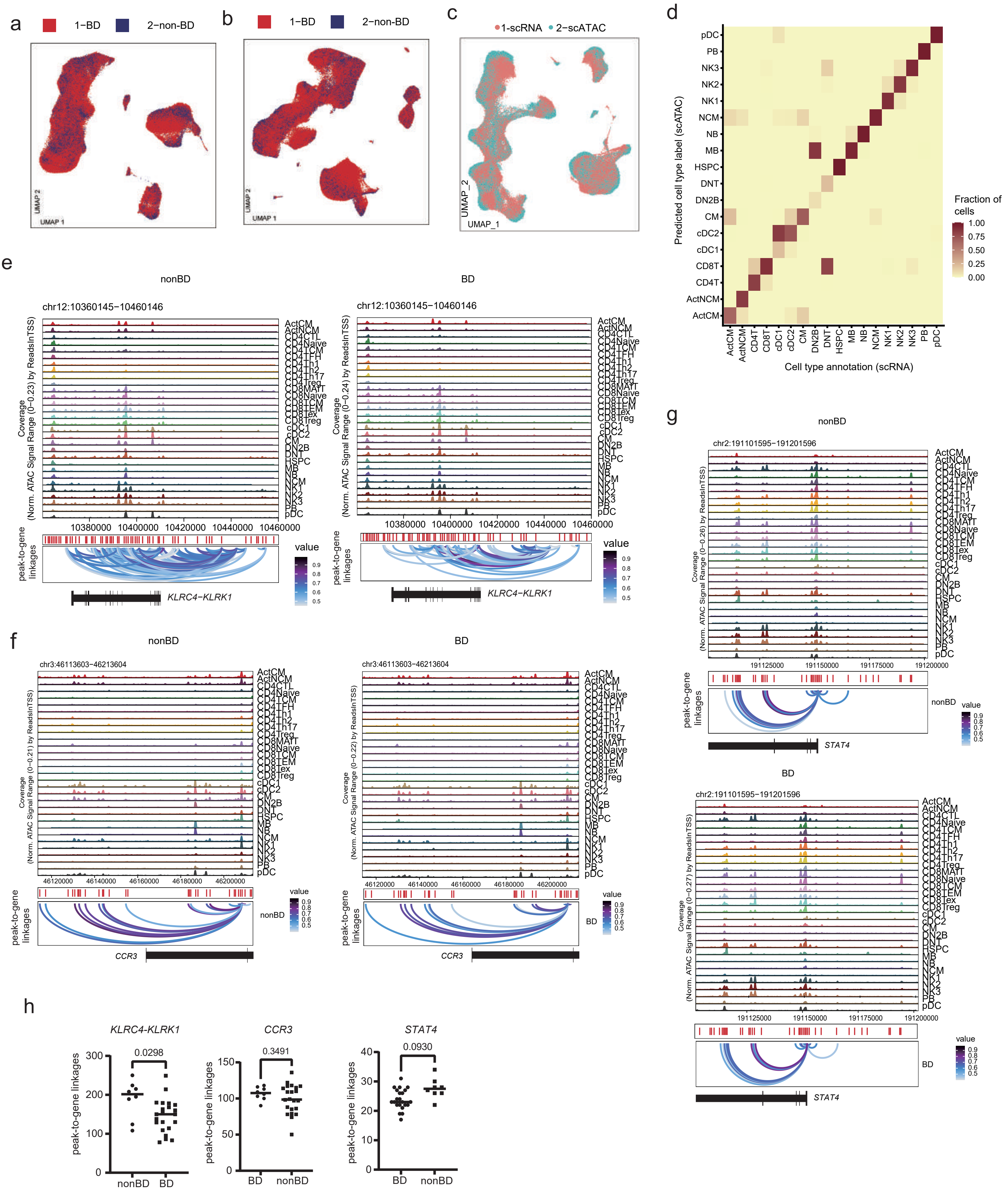
a
1−BD
2−non−BD
UMAP 1
UMAP 2
b
1−BD
2−non−BD
c
1-scRNA
2-scATAC
UMAP_1
UMAP_2
d
Predicted cell type label (scATAC)
Cell type annotation (scRNA)
Fraction of cells
e
nonBD
BD
chr12:10360145−10460146
KLRC4−KLRK1
peak-to-gene linkages
value
f
chr3:46113603−46213604
CCR3
g
chr2:191101595−191201596
STAT4
h
KLRC4-KLRK1
0.0298
CCR3
0.3491
STAT4
0.0930
peak-to-gene linkages

Supplementary Figure 13. Applications of Seurat to integrate scATAC-seq and scRNA-seq cells from BD and non-BD individuals.

a. UMAP projection of scRNA-seq dataset colored by different clinical states (non-BD and BD) as indicated. (non-BD=46,968 cells, nBD=105,736 cells). **b.** UMAP projection of scATAC-seq dataset colored by different clinical states (non-BD and BD) as indicated. (non-BD=68,275 cells, nBD=203,838 cells). **c.** UMAP projection of integrated scATAC-seq and scRNA-seq dataset colored by different cell types as indicated. **d.** Confusion matrix showing the cell type assignment of scATAC-seq and scRNA-seq dataset achieved by the Seurat's canonical correlation analysis. The color intensity represents the fraction of the assigned cells per cell type. **e.** Cis-regulatory architecture in PBMCs (left panel: non-BD groups; right panel: BD groups): *KLRC4-KLRK1*. Only connections originating in the loci with peak-to-gene accessibility above 0.4 are shown. **f.** Cis-regulatory architecture in PBMCs (left panel: non-BD groups; right panel: BD groups): *CCR3*. Only connections originating in the loci with peak-to-gene accessibility above 0.4 are shown. **g.** Cis-regulatory architecture in PBMCs (left panel: non-BD groups; right panel: BD groups): *STAT4*. Only connections originating in the loci with peak-to-gene accessibility above 0.4 are shown. **h.** Differences in the numbers of peak-to-gene linkages of each donor in BD and nonBD groups. The p values were calculated using two-sided Wilcoxon rank-sum test. The peak-to-gene accessibility above 0.4 are calculated in each donor. The horizontal lines denote median. All data are aligned and annotated to hg38 reference genome.

a

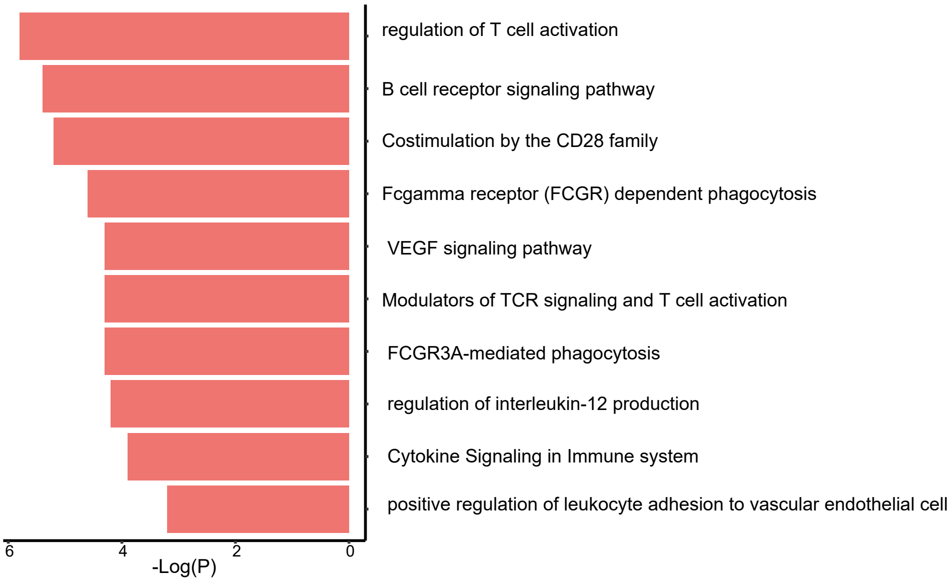
regulation of T cell activation
B cell receptor signaling pathway
Costimulation by the CD28 family
Fcgamma receptor (FCGR) dependent phagocytosis
VEGF signaling pathway
Modulators of TCR signaling and T cell activation
FCGR3A-mediated phagocytosis
regulation of interleukin-12 production
Cytokine Signaling in Immune system
positive regulation of leukocyte adhesion to vascular endothelial cell
6
4
2
0
-Log(P)

b

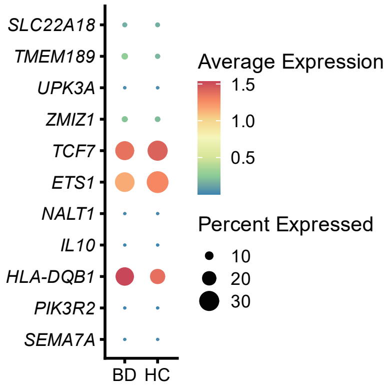
SLC22A18
TMEM189
UPK3A
ZMIZ1
TCF7
ETS1
NALT1
IL10
HLA-DQB1
PIK3R2
SEMA7A
BD
HC
Average Expression
1.5
1.0
0.5
Percent Expressed
10
20
30

Supplementary Figure 14. Applications of FigR to predict gene regulatory network (GRN).

a. Representative GO terms and KEGG pathways among DORC genes(n=202). **b.** Dotplot of DORC genes between BD/non-BD comparison groups. All data are aligned and annotated to hg38 reference genome.
