# Supplementary material for: Single-cell chromatin accessibility and transcriptomic characterization of Behcet’s disease
Source: Commun Biol. 2023 Oct 17;6:1048. doi: 10.1038/s42003-023-05420-x (PMC10582193; doi:10.1038/s42003-023-05420-x)
Supplement: Supplementary file 3 — Description of Additional Supplementary Files [file 42003_2023_5420_MOESM3_ESM.docx]

Description of Additional Supplementary Files

**File name: Supplementary Data 1**

Description: Subjects' information.

**File name: Supplementary Data 2**

Description: The detailed cell counts and frequencies of cells in scATAC-seq and scRNA-seq datasets.

**File name: Supplementary Data 3**

Description: The statistical results of TF footprint analysis in immune cells.

**File name: Supplementary Data 4**

Description: The detailed DEGs results using Seurat in T cell subsets among non-BD and BD groups.

**File name: Supplementary Data 5**

Description: The detailed DEGs results using Muscat in T cell subsets among non-BD and BD groups.

**File name: Supplementary Data 6**

Description: The detailed GO analysis results in scRNA-seq datasets.

**File name: Supplementary Data 7**

Description: The detailed DEGs results using Seurat and Muscat in NK, BC, and DC subsets among non-BD and BD groups.

**File name: Supplementary Data 8**

Description: The detailed DEGs results using Seurat in monocyte subsets.

**File name: Supplementary Data 9**

Description: The average gene expression and statistical analysis of *KLRC4-KLRK1* in BD and non-BD individuals.

**File name: Supplementary Data 10**

Description: DORC genes.

**File name: Supplementary Data 11**

Description: Putative gene regulatory network in BD.
