# Supplementary material for: Single-cell chromatin accessibility and transcriptomic characterization of Behcet’s disease
Source: Commun Biol. 2023 Oct 17;6:1048. doi: 10.1038/s42003-023-05420-x (PMC10582193; doi:10.1038/s42003-023-05420-x)
Supplement: Supplementary file 12 — Reporting Summary [file 42003_2023_5420_MOESM12_ESM.pdf]

nature portfolio

Corresponding author(s): Yingfeng Zheng

Last updated by author(s): 2023/09/19

Reporting Summary

Nature Portfolio wishes to improve the reproducibility of the work that we publish. This form provides structure for consistency and transparency in reporting. For further information on Nature Portfolio policies, see our Editorial Policies and the Editorial Policy Checklist.

Statistics

For all statistical analyses, confirm that the following items are present in the figure legend, table legend, main text, or Methods section.

n/a	Confirmed	
☐	☒	The exact sample size (*n*) for each experimental group/condition, given as a discrete number and unit of measurement
☐	☒	A statement on whether measurements were taken from distinct samples or whether the same sample was measured repeatedly
☐	☒	The statistical test(s) used AND whether they are one- or two-sided<br>*Only common tests should be described solely by name; describe more complex techniques in the Methods section.*
☒	☐	A description of all covariates tested
☐	☒	A description of any assumptions or corrections, such as tests of normality and adjustment for multiple comparisons
☐	☒	A full description of the statistical parameters including central tendency (e.g. means) or other basic estimates (e.g. regression coefficient) AND variation (e.g. standard deviation) or associated estimates of uncertainty (e.g. confidence intervals)
☐	☒	For null hypothesis testing, the test statistic (e.g. *F*, *t*, *r*) with confidence intervals, effect sizes, degrees of freedom and *P* value noted<br>*Give P values as exact values whenever suitable.*
☒	☐	For Bayesian analysis, information on the choice of priors and Markov chain Monte Carlo settings
☒	☐	For hierarchical and complex designs, identification of the appropriate level for tests and full reporting of outcomes
☒	☐	Estimates of effect sizes (e.g. Cohen's *d*, Pearson's *r*), indicating how they were calculated

Our web collection on statistics for biologists contains articles on many of the points above.

Software and code

Policy information about availability of computer code

Data collection	Cell Ranger 3.1.0 - Barcode Identification, Alignment, Filter, Deduplication
Data analysis	All custom code used in this work is desposited in Zenodo https://doi.org/10.5281/zenodo.834834

For manuscripts utilizing custom algorithms or software that are central to the research but not yet described in published literature, software must be made available to editors and reviewers. We strongly encourage code deposition in a community repository (e.g. GitHub). See the Nature Portfolio guidelines for submitting code & software for further information.

Data

Policy information about availability of data

All manuscripts must include a data availability statement. This statement should provide the following information, where applicable:

- Accession codes, unique identifiers, or web links for publicly available datasets
- A description of any restrictions on data availability
- For clinical datasets or third party data, please ensure that the statement adheres to our policy

The scRNA-seq, scATAC-seq and bulk RNA-seq data analyzed in the article are available from the corresponding author upon request under the Project Accession No. PRJCA004696 and the GSA Accession No. HRA004778 in https://ngdc.cncb.ac.cn/gsa-human/.

Human research participants

Policy information about studies involving human research participants and Sex and Gender in Research.

Reporting on sex and gender	The sex and gender were not considered in this study. The non-BD group consisted of eight individuals, 4 men and 4 women, with an average age of 46.8 years (Supplementary Table 1). The BD patient cohort (Supplementary Table 1) comprised 13 men and 10 women, with an average age of 33.3 years.
Population characteristics	See above.
Recruitment	Written informed consent was obtained from all participating individuals, who were recruited from Zhongshan Ophthalmic Center.
Ethics oversight	The Ethics Committee of Zhongshan Ophthalmic Center (Guangzhou, China, 2019KYPJ114) approved this study, which followed the relevant ethical regulations for human research participants according to the Declaration of Helsinki.

Note that full information on the approval of the study protocol must also be provided in the manuscript.

Field-specific reporting

Please select the one below that is the best fit for your research. If you are not sure, read the appropriate sections before making your selection.

☒ Life sciences ☐ Behavioural & social sciences ☐ Ecological, evolutionary & environmental sciences

For a reference copy of the document with all sections, see nature.com/documents/nr-reporting-summary-flat.pdf

Life sciences study design

All studies must disclose on these points even when the disclosure is negative.

Sample size	Exclusion criteria for the study included comorbid conditions such as cancer, immunocompromising disorders, hypertension, diabetes, and steroid use. The non-BD group consisted of eight individuals, 4 men and 4 women, with an average age of 46.8 years (Supplementary Table 1). The BD patient cohort (Supplementary Table 1) comprised 13 men and 10 women, with an average age of 33.3 years.
Data exclusions	For scATAC-seq and scRNA-seq experiments, the details of our data quality control procedures are provided in the methods section of the manuscript.
Replication	All results presented in manuscript were reliably reproduced.
Randomization	No randomization in this study.
Blinding	No blinding in this study.

Reporting for specific materials, systems and methods

We require information from authors about some types of materials, experimental systems and methods used in many studies. Here, indicate whether each material, system or method listed is relevant to your study. If you are not sure if a list item applies to your research, read the appropriate section before selecting a response.

Materials & experimental systems

n/a	Involved in the study	
☒	☐	Antibodies
☒	☐	Eukaryotic cell lines
☒	☐	Palaeontology and archaeology
☒	☐	Animals and other organisms
☐	☐	Clinical data
☒	☐	Dual use research of concern

Methods

n/a	Involved in the study	
☒	☐	ChIP-seq
☒	☐	Flow cytometry
☒	☐	MRI-based neuroimaging

Clinical data

Policy information about clinical studies

All manuscripts should comply with the ICMJE guidelines for publication of clinical research and a completed CONSORT checklist must be included with all submissions.

Clinical trial registration	NCT04101604
Study protocol	2019KYPJ114
Data collection	The study was approved by the Ethics Committee of Zhongshan Ophthalmic Center (Guangzhou, China, 2019KYPJ114), and followed the relevant ethical regulations for human research participants according to the Declaration of Helsinki. Written informed consent was obtained from all participating individuals, who were recruited from Zhongshan Ophthalmic Center.
Outcomes	Patients who diagnosed based on the revised diagnostic criteria established by the 2013 International Criteria for BD.
